# The InBIO Barcoding Initiative Database: DNA barcodes of Portuguese Hemiptera 01

**DOI:** 10.3897/BDJ.9.e65314

**Published:** 2021-07-29

**Authors:** Pedro Sousa, José Manuel Grosso-Silva, Rui Andrade, Cátia Chaves, Joana Pinto, Joana Paupério, Pedro Beja, Sónia Ferreira

**Affiliations:** 1 CIBIO/InBIO, Centro de Investigação em Biodiversidade e Recursos Genéticos, Universidade do Porto, Campus Agrário de Vairão, 4485-661 Vairão, Vila do Conde, Portugal CIBIO/InBIO, Centro de Investigação em Biodiversidade e Recursos Genéticos, Universidade do Porto, Campus Agrário de Vairão, 4485-661 Vairão Vila do Conde Portugal; 2 Museu de História Natural e da Ciência da Universidade do Porto, Porto, Portugal Museu de História Natural e da Ciência da Universidade do Porto Porto Portugal; 3 Rua Calouste Gulbenkian 237 4H3 4050-145, Porto, Portugal Rua Calouste Gulbenkian 237 4H3 4050-145 Porto Portugal; 4 CIBIO/InBIO, Centro de Investigação em Biodiversidade e Recursos Genéticos, Instituto Superior de Agronomia, Universidade de Lisboa, Tapada da Ajuda, 1349-017, Lisboa, Portugal CIBIO/InBIO, Centro de Investigação em Biodiversidade e Recursos Genéticos, Instituto Superior de Agronomia, Universidade de Lisboa, Tapada da Ajuda, 1349-017 Lisboa Portugal

**Keywords:** Hemiptera, occurrence records, continental Portugal, DNA barcode, COI

## Abstract

**Background:**

The InBIO Barcoding Initiative (IBI) Hemiptera 01 dataset contains records of 131 specimens of Hemiptera. Most specimens have been morphologically identified to species or subspecies level and represent 88 species in total. The species of this dataset correspond to about 7.3% of continental Portuguese hemipteran species diversity. All specimens were collected in continental Portugal. Sampling took place from 2015 to 2019 and specimens are deposited in the IBI collection at CIBIO, Research Center in Biodiversity and Genetic Resources.

**New information:**

This dataset increases the knowledge on the DNA barcodes and distribution of 88 species of Hemiptera from Portugal. Six species, from five different families, were new additions to the Barcode of Life Data System (BOLD), with another twenty five species barcodes' added from under-represented taxa in BOLD. All specimens have their DNA barcodes publicly accessible through BOLD online database and the distribution data can be accessed through the Global Biodiversity Information Facility (GBIF). *Eutettix variabilis* and *Fieberiella florii* are recorded for the first time for Portugal and *Siphanta acuta*, an invasive species, previously reported from the Portuguese Azores archipelago, is recorded for the first time for continental Portugal.

## Introduction

Hemiptera is the most diverse order of non-holometabolan insects, with more than 107,000 described species (Henry 2017, Bartlett et al. 2018, Hardy 2018), being second only to the four so-called “megadiverse” holometabolan orders (Coleoptera, Lepidoptera, Diptera, and Hymenoptera), which include over 150,000 described species each (Zhang 2013). Hemipterans are among the most abundant and widespread insects on land and in freshwater habitats (Andersen 1999). The Hemiptera, or true bugs, have piercing‐sucking mouthparts that constrain them to feed on liquid food (Schuh and Slater 1995, Scudder 2017, Panfilio and Angelini 2018). The primary feeding habit of Hemiptera is herbivory but the order also includes numerous carnivores, scavengers, hematophages and some necrophages (Forero 2008, Gullan and Cranston 2014). As a result their ecological role is strongly linked to their trophic interaction with plants, several species are among the most important crop pests (Schuh and Slater 1995, Schaefer and Panizzi 2000, Dietrich 2009, Gullan and Martin 2009, Scudder 2017). A few hematophagous hemipterans in the subfamily Triatominae (Reduviidae) have a direct impact on human health as vectors of Chagas disease (Balczun et al. 2012).

In continental Portugal, the knowledge about the order Hemiptera is fragmentary and heterogeneous. The latest diversity estimate was close to 1,100 species (Grosso-Silva 2003), but the description of new species (e.g., Emeljanov and Drosopoulos 2004, Ribes and Baena 2006, Sanchez et al. 2006), as well as the detection of previously unrecorded ones (e.g. Grosso-Silva 2004, Hollier 2005, Goula and Mata 2011, Baena and Zuzarte 2012, Foster 2019, Grosso-Silva and Ferreira 2020) lead to an estimated number of more than 1,200 species to date. However, additional studies are needed to validate the distribution of the species in general. Furthermore, the introduction or expansion of alien species from nearby areas has also occurred regularly (e.g. Valente et al. 2004, Franco et al. 2011, Sánchez 2011, Borges et al. 2013, Garcia et al. 2013, Bella 2014, Grosso-Silva et al. 2020).

DNA barcoding is a standard molecular biology method for species identification based on the sequencing of a short mitochondrial DNA sequence that is then compared to a library of known sequences (Hebert et al. 2003). The construction of such libraries is an essential step in the process that requires the morphological identification of specimens to establish a baseline for comparisons (Kress et al. 2015, Ferreira et al. 2018). Open libraries of DNA barcodes exist, namely the Barcode of Life Data System (BOLD), but they are not comprehensive yet, especially in regions of high diversity or endemicity. Furthermore, regional variation in species genetic variability can confound identification results (Phillips et al. 2019). DNA barcodes can be used as a discovery step, on a two-step approach of species delimitation (e.g. Rannala 2015), but also combined with ecological traits (Kress et al. 2015), greatly contributing to the solution of the taxonomic impediment problem in Biology (e.g. Riedel et al. 2013, Kekkonen and Hebert 2014). DNA barcodes usefulness has rapidly extended beyond organism and species identification; they are increasingly used in ecological and biological conservation studies, as well as in forensic applications, such as food source identification (Pečnikar and Buzan 2013, Kress et al. 2015, DeSalle and Goldstein 2019). DNA barcoding has been successfully applied to the Hemiptera (e.g. Jung et al. 2011, Park et al. 2011, Raupach et al. 2014, Havemann et al. 2018, Govender and Willows-Munro 2019), with identification success rates of 80% to 100%. It is especially useful to identify immature and female individuals’ (e.g. Raupach et al. 2014, Havemann et al. 2018), which may not be reliably identified through morphological characters, or in areas where diversity remains poorly known (e.g. Govender and Willows-Munro 2019). DNA barcoding as also highlighted the existence of cryptic diversity and the need for taxonomic revisions of certain taxa (e.g. Jung et al. 2011, Park et al. 2011, Raupach et al. 2014, Havemann et al. 2018, Govender and Willows-Munro 2019).

In this context, Portuguese biodiversity is still underestimated and undersampled, although being part of the westernmost portion of the Mediterranean hotspot of biodiversity. The paucity of genetic data on Portuguese biodiversity led to the creation of a DNA barcoding initiative by the Research Network in Biodiversity and Evolutionary Biology - InBIO. The InBIO Barcoding Initiative (IBI) makes use of High-Throughput Sequencing technologies to construct a reference collection of morphologically identified Portuguese specimens and respective DNA barcodes. Within IBI, invertebrates, and insects in particular, are prioritied, given their large contribution to overall biodiversity and ecosystems (e.g. Weisser and Siemann 2004, Losey and Vaughan 2006, Mata et al. 2016, Silva et al. 2019) and the clear shortage of DNA barcodes available in public databases (e.g. Corley and Ferreira 2017, Corley et al. 2017, Ferreira et al. 2019, Weigand et al. 2019).

The IBI Hemiptera 01 dataset contains records of 131 specimens of Hemiptera collected in continental Portugal, all of which were identified to species level, mostly through morphological identification, for a total of 88 species and one additional subspecies. This dataset is the first IBI dataset on Hemiptera and is part of the ongoig IBI database public releases in both the Global Biodiversity Information Facility (GBIF) and the Barcode of Life Data System (BOLD) (e.g. Ferreira et al. 2020a, Ferreira et al. 2020b). We have included in this dataset the barcodes of all identified Hemiptera specimens in IBI up to December 2020. Overall, this paper contributes to the open dissemination and sharing of the distribution records and DNA barcodes of Hemiptera specimens that are part of our reference collection, to increase the available public information on a group of Portuguese Invertebrates.

## General description

### Purpose

This dataset aims to provide a first contribution to an authoritative DNA barcode sequences library for Portuguese Hemiptera. Such a library aims to enable DNA-based identification of species for both traditional molecular studies and DNA-metabarcoding studies. Furthermore, it constitutes an important resource for taxonomic research on Portuguese Hemiptera and its distribution.

### Additional information

A total of 131 specimens of hemipterans were collected and DNA Barcodes (Suppl. materials 1, 2). Fig. 1 illustrates examples of the diversity of species that are part of the dataset of distribution data and DNA barcodes of Portuguese Hemiptera 01. All sequences of cytochrome *c* oxidase I (COI) DNA barcodes are 658 base pairs (bp) long, except for one with 418 bp. From the 88 species barcoded, six (7%) from five families are new to the DNA barcode database BOLD at the moment of its release (January 2021, marked with * in Species field of Table 1). Twenty-five additional taxa (28%) from 17 families were already represented in BOLD with less than 10 DNA barcode sequences (marked with " in Species field of Table 1). A few noteworthy species are included in the dataset. The record of the species *Eutettix variabilis* Hepner, 1942 is, to the best of our knowledge, the first record published for Portugal. European records for this north American species (Metcalf 1967) exist online (e.g. http://boldsystems.org/index.php/Taxbrowser_Taxonpage?taxon=+Eutettix+variabilis&searchTax=Search+Taxonomy; all European records in BOLD are based on genetic identifications). The species *Fieberiella florii* Stål, 1864, a vector for phytoplasmas, is also recorded for Portugal for the first time, with a few records known for Spain (e.g. Aguin-Pombo et al. 2007). Another important result is the record of the invasive species *Siphanta acuta* (Walker, 1851), recorded here for the first time for continental Portugal, although it has been previously reported from the São Miguel Island in the Azores Archipelago (Borges et al. 2013). Moreover, *Stictopleurus punctatonervosus*, first recorded from Murtosa (Aveiro) (Valcárcel and Prieto Piloña 2021), is recorded for the second time for Portugal.

## Project description

### Personnel

Pedro Beja (project coordinator), Sónia Ferreira (taxonomist and IBI manager), Joana Paupério (IBI manager), Pedro Sousa (taxonomist, project technician), Cátia Chaves (project technician), Joana Pinto (project technician), all affiliated to CIBIO-InBIO, University of Porto, José Manuel Grosso-Silva (taxonomist), affiliated to the MHNC-UP, University of Porto and Rui Andrade (taxonomist), independent researcher.

## Sampling methods

### Study extent

Continental Portugal.

### Sampling description

**Figure 2.**
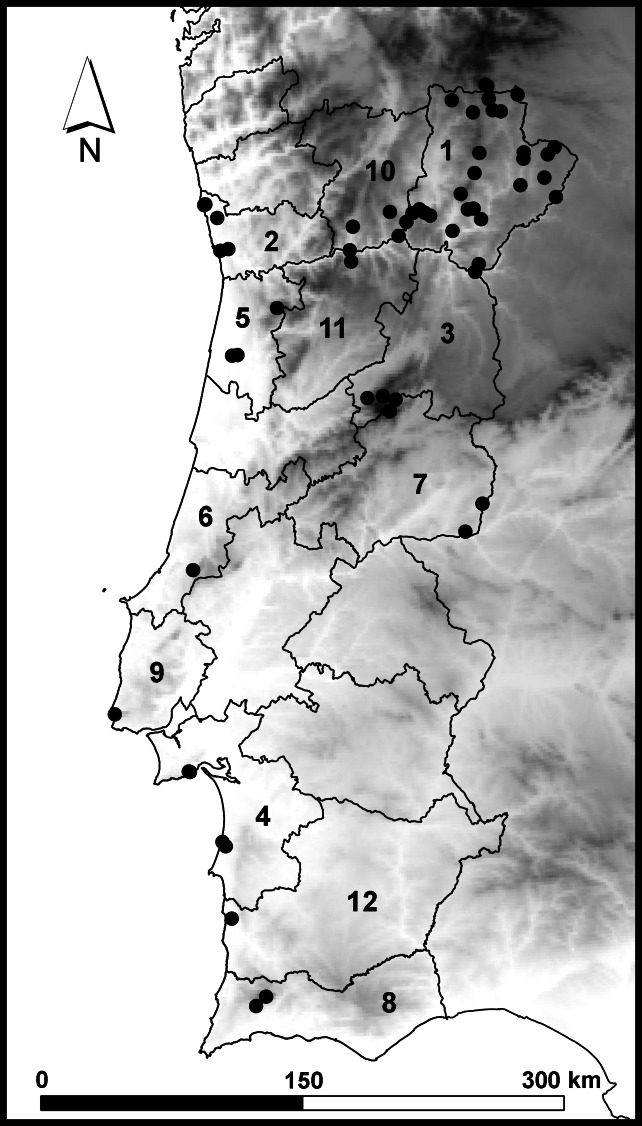
Map of the localities where Hemiptera samples were collected in continental Portugal. Portuguese Districts are also represented, with those referred in Table 2 numbered as follows: 1 - Bragança, 2 - Porto, 3 - Guarda, 4 - Setúbal, 5 - Aveiro, 6 - Leiria, 7 - Castelo Branco, 8 - Faro, 9 - Lisboa, 10 - Vila Real, 11 - Viseu, 12 - Beja.

The studied material was collected in 60 different localities from continental Portugal, almost half of which (47%) belong to the Bragança District (Fig. 2, Table 2). Two specimens were integrated in the IBI reference collection without further sampling information available besides being collected in Portugal. Sampling was conducted between 2015 and 2019 in a wide range of habitats, by direct search of specimens or by sweeping the vegetation. Collected specimens were examined using a stereoscopic microscope and stored in 96% ethanol for downstream molecular analysis. Morphological identification was performed, based on keys and descriptions from literature (Suppl. material 3). DNA extraction and sequencing followed the general pipeline used in the InBIO Barcoding Initiative. Genomic DNA was extracted from leg tissue using EasySpin Genomic DNA Tissue Kit (Citomed) following the manufacturer’s protocol. The mitochondrial cytochrome *c* oxidase I (COI) barcoding fragment was amplified as two overlapping fragments (LC and BH), using two sets of primers: LCO1490 (Folmer et al. 1994) + Ill_C_R and Ill_B_F (Shokralla et al. 2015) + HCO2198 (Folmer et al. 1994), respectively. The COI gene (Folmer region), was then sequenced in a MiSeq benchtop system. OBITools (Boyer et al. 2015) was used to process the initial sequences which were then assembled into a single 658 bp fragment using Geneious 9.1.8. (https://www.geneious.com).

### Quality control

All DNA barcode sequences were compared against the BOLD database and the 99 top results were inspected in order to detect possible problems due to contaminations or misidentifications. Prior to GBIF submission, data were checked for errors and inconsistencies with OpenRefine 3.3 (http://openrefine.org).

### Step description

1. Specimens were collected in 60 different localities of continental Portugal. Fieldwork was carried out between 2015 and 2019.
2. Specimens were collected during fieldwork by direct search of specimens or by sweeping the vegetation with a hand-net and preserved in 96% alcohol. Captured specimens were deposited in the IBI reference collection at CIBIO (Research Center in Biodiversity and Genetic Resources).
3. Specimens were morphologically identified with the assistance of stereoscopic microscopes (Leica MZ12, 8x to 100x; Olympus SZX16, 7x to 115x) and using the available literature (Suppl. material 3). A subset (23%) was identified using the BOLD Identification Engine directly.
4. DNA barcodes were sequenced from all specimens. For this, one leg was removed from each individual, DNA was then extracted and a 658 bp COI DNA barcode fragment was amplified and sequenced. For one specimen of *Ceraleptus lividus*, only a 418 bp fragment was sequenced. DNA extracts were deposited in the IBI collection.
5. All obtained sequences were submitted to BOLD and GenBank databases and, to each sequenced specimen, the morphological identification, when available, was contrasted with the results of the BLAST of the newly-generated DNA barcodes in the BOLD Identification Engine.
6. Prior to submission to GBIF, data were checked for errors and inconsistencies with OpenRefine 3.3 (http://openrefine.org/).

## Geographic coverage

### Description

Continental Portugal .

### Coordinates

37.257 and 41.979 Latitude; -9.465 and -6.344 Longitude.

## Taxonomic coverage

### Description

**Figure 3.**
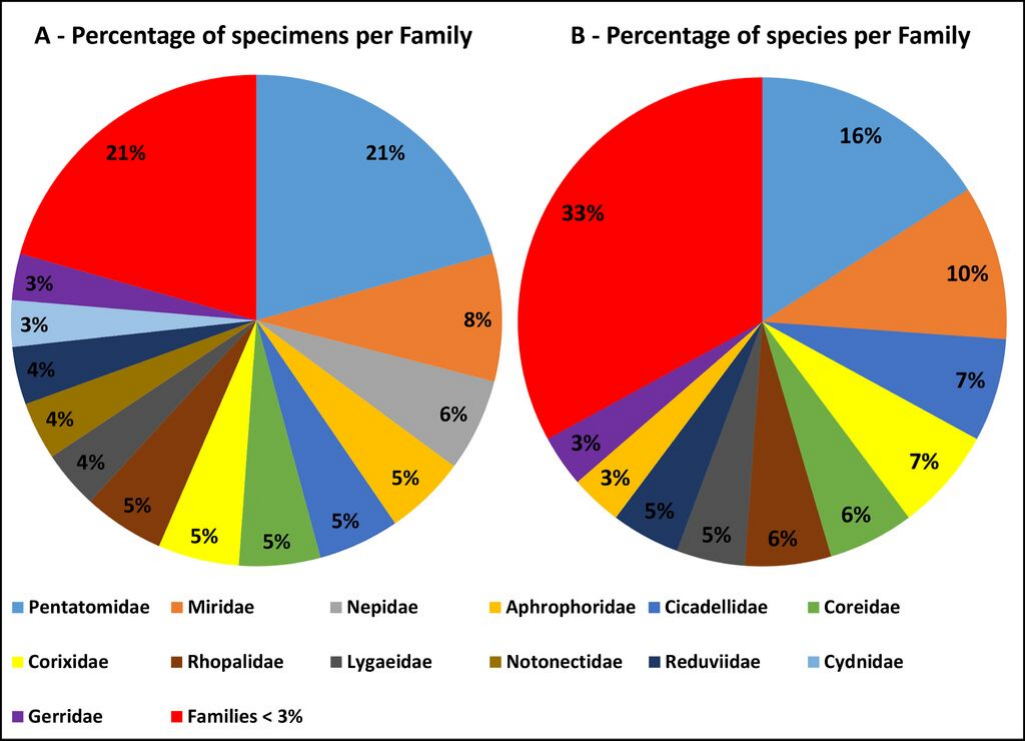
Distribution of specimens (A) and species (B), in percentage, per Hemiptera family present in the dataset. Families representing less than 3% of the total specimens/species are represented together in the respective graph.

This dataset is composed of data relating to 131 Hemiptera specimens. All specimens were determined to species level, with three specimens further identifed to subspecies level. Overall, 88 species are represented in the dataset. These species belong to 30 families. The Pentatomidae family accounts for 21% of the total collected specimens (Fig. 3A) and no other family accounts for more than 8%. The Pentatomidae and Miridae families combined account for 26% of the total taxa represented (Fig. 3B) and no other family accounts for more than 7%. Eleven families are represented by a single taxon and nine by two taxa.

### Taxa included

**Table:** 

Rank	Scientific Name	
kingdom	Animalia	
phylum	Arthropoda	
subphylum	Hexapoda	
class	Insecta	
order	Hemiptera	
superorder	Auchenorrhyncha	
superorder	Heteroptera	
family	Acanthosomatidae	
family	Alydidae	
family	Aphrophoridae	
family	Aradidae	
family	Berytidae	
family	Cercopidae	
family	Cicadellidae	
family	Cicadidae	
family	Coreidae	
family	Corixidae	
family	Cydnidae	
family	Delphacidae	
family	Dictyopharidae	
family	Flatidae	
family	Gerridae	
family	Hydrometridae	
family	Lygaeidae	
family	Membracidae	
family	Miridae	
family	Nabidae	
family	Nepidae	
family	Notonectidae	
family	Pentatomidae	
family	Potamocoridae	
family	Pyrrhocoridae	
family	Reduviidae	
family	Rhopalidae	
family	Rhyparochromidae	
family	Stenocephalidae	
family	Thaumastocoridae	

## Temporal coverage

**Data range:** 2015-3-16 – 2019-9-20.

### Notes

The sampled material was collected in the period from 16 March 2015 to 20 September 2019.

## Usage licence

### Usage licence

Other

### IP rights notes

Creative Commons Attribution 4.0 International (CC BY 4.0)

## Data resources

### Data package title

The InBIO Barcoding Initiative Database: Hemiptera 01

### Resource link

http://www.boldsystems.org/index.php/Public_SearchTerms?query=DS-IBIHP01

### Number of data sets

1

### Data set 1.

#### Data set name

DS-IBIHP01 IBI Hemiptera 01

#### Data format

dwc, xml, tsv, fasta

#### Number of columns

37

#### Description

The InBIO Barcoding Initiative Database: Hemiptera 01 dataset can be downloaded from BOLD (dx.doi.org/10.5883/DS-IBIHP01) in different formats (records as dwc, xml or tsv and sequences as fasta files). All records are also searchable within BOLD, using the search function of the platform.

The InBIO Barcoding Initiative will continue to sequence Hemiptera for the BOLD database, with the ultimate goal of achieving a comprehensive coverage of the Portuguese Hemiptera fauna. The version of the dataset, at the time of the writing of the manuscript, is included as Suppl. materials 1, 2, 4 in the form of two text files with specimen data information, as downloaded from BOLD and GBIF (the latter in Darwin Core Standard format) and one fasta file containing all sequences as downloaded from BOLD.

It should be noted that the BOLD database is not strictly compliant with the Darwin Core Standard (DwC) format and, as such, the file downloadable from BOLD (Suppl. material 1) is not in the standard DwC. For a proper DwC formatted file, see http://ipt.gbif.pt/ipt/resource?r=ibi_hemiptera_01&amp;v=1.0 (Suppl. material 2).

Column labels below follow the labels downloaded in the tsv format from BOLD. Columns with no content in our dataset are left out in the list below.

**Data set 1.** 

Column label	Column description
processid	Unique identifier for the sample
sampleid	Identifier for the sample being sequenced, i.e. IBI catalogue number at Cibio-InBIO, Porto University. Often identical to the "Field ID" or "Museum ID"
recordID	Identifier for specimen assigned in the field
catalognum	Catalogue number
fieldnum	Field number
institution_storing	The full name of the institution that has physical possession of the voucher specimen
bin_uri	Barcode Index Number system identifier
phylum_taxID	Phylum taxonomic numeric code
phylum_name	Phylum name
class_taxID	Class taxonomic numeric code
class_name	Class name
order_taxID	Order taxonomic numeric code
order_name	Order name
family_taxID	Family taxonomic numeric code
family_name	Family name
subfamily_taxID	Subfamily taxonomic numeric code
subfamily_name	Subfamily name
genus_taxID	Genus taxonomic numeric code
genus_name	Genus name
species_taxID	Species taxonomic numeric code
species_name	Species name
identification_provided_by	Full name of primary individual who assigned the specimen to a taxonomic group
identification_method	The method used to identify the specimen
voucher_status	Status of the specimen in an accessioning process (BOLD controlled vocabulary)
tissue_type	A brief description of the type of tissue or material analysed
collectors	The full or abbreviated names of the individuals or team responsible for collecting the sample in the field
lifestage	The age class or life stage of the specimen at the time of sampling
sex	The sex of the specimen
lat	The geographical latitude (in decimal degrees) of the geographic centre of a location
lon	The geographical longitude (in decimal degrees) of the geographic centre of a location
elev	Elevation of sampling site (in metres above sea level)
country	The full, unabbreviated name of the country where the organism was collected
province_state	The full, unabbreviated name of the province ("Distrito" in Portugal) where the organism was collected
region	The full, unabbreviated name of the municipality ("Concelho" in Portugal) where the organism was collected
exactsite	Additional name/text description regarding the exact location of the collection site relative to a geographic relevant landmark
subspecies_taxID	Subspecies taxonomic numeric code
subspecies_name	Subspecies name

## Supplementary Material

FF5FEB5D-611B-5FD1-8453-EC3A4A5A29C8 10.3897/BDJ.9.e65314.suppl1 Supplementary material 1 IBI - Hemiptera 01 library - Specimen details Data type Specimen data records Brief description The file includes information about all records in BOLD for the IBI - Hemiptera 01 library. It contains collecting and identification data. The data are as downloaded from BOLD, without further processing. File: oo_557157.txt https://binary.pensoft.net/file/557157 Pedro Sousa, José Manuel Grosso-Silva, Rui Andrade, Pedro Beja, Sónia Ferreira

AC3D7D5D-F4DA-5D18-917E-32982BFB9114 10.3897/BDJ.9.e65314.suppl2 Supplementary material 2 IBI - Hemiptera 01 library - Specimen details - Darwin Core Standard Data type Specimen data records in Darwin Core Standard format Brief description The file includes information about all records in GBIF for the IBI - Hemiptera 01 library. It contains collecting and identification data. The data are as downloaded from GBIF, without further processing. File: oo_557158.txt https://binary.pensoft.net/file/557158 Pedro Sousa, José Manuel Grosso-Silva, Rui Andrade, Pedro Beja, Sónia Ferreira

F9FD2F63-D47D-5739-B281-F1B0FAB1B32D 10.3897/BDJ.9.e65314.suppl3 Supplementary material 3 References used for morphological identification Data type References Brief description References used for morphological identification. File: oo_511642.txt https://binary.pensoft.net/file/511642 José Manuel Grosso-Silva, Pedro Sousa

FB57D8C9-0C10-533C-BF8F-BAD23DF5F6C0 10.3897/BDJ.9.e65314.suppl4 Supplementary material 4 IBI- Hemiptera 01 library - DNA sequences Data type Specimen genomic data, DNA sequences Brief description COI sequences in fasta format. Each sequence is identified by the BOLD ProcessID, species name, genetic marker name and GenBank accession number, all separated by a vertical bar. The data are as downloaded from BOLD. File: oo_511641.fas https://binary.pensoft.net/file/511641 Pedro Sousa, Cátia Chaves, Joana Pinto, Joana Paupério, Pedro Beja, Sónia Ferreira

## Figures and Tables

**Figure 1a.**
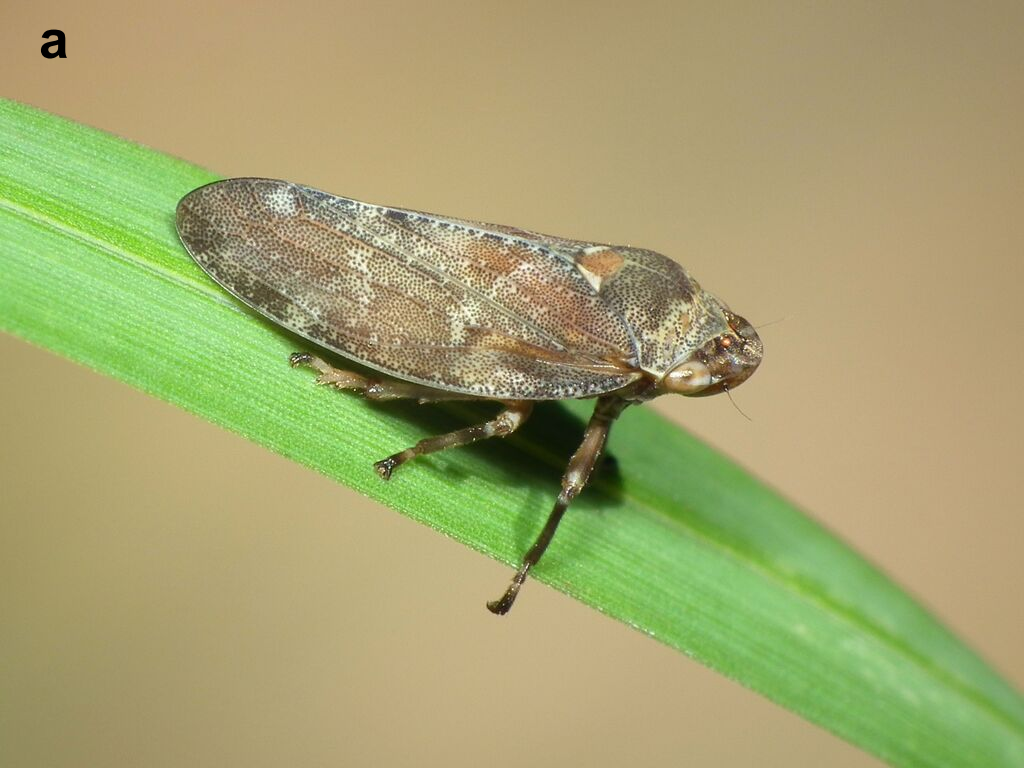
*Aphrophora corticea* - BIN URI: BOLD:ACT0928

**Figure 1b.**
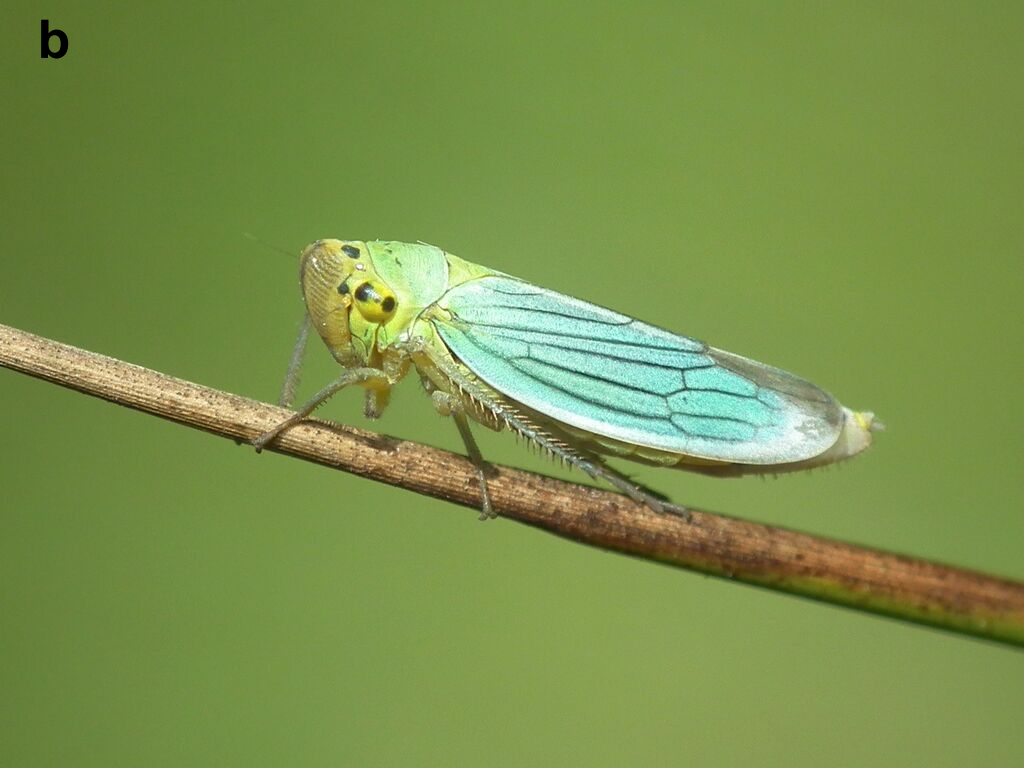
*Cicadella viridis* - BIN URI: BOLD:ACB8347

**Figure 1c.**
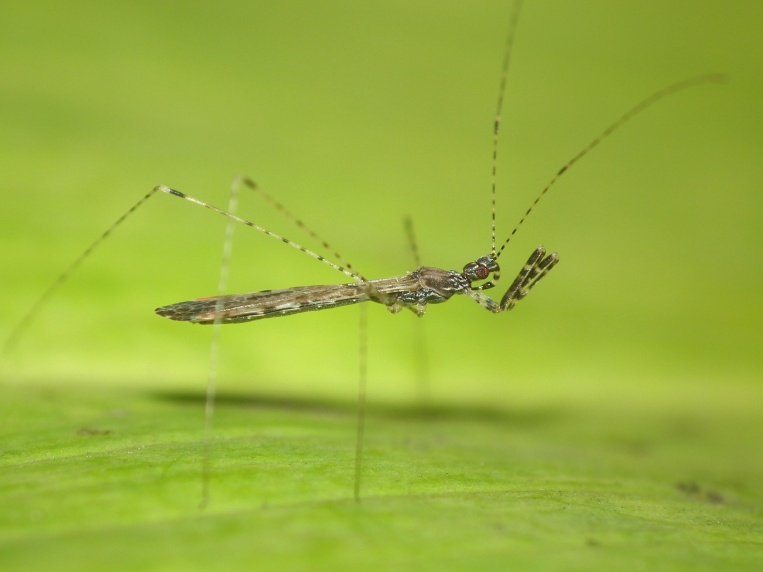
*Empicoris rubromaculatus* - BIN URI: BOLD:ACN7256

**Figure 1d.**
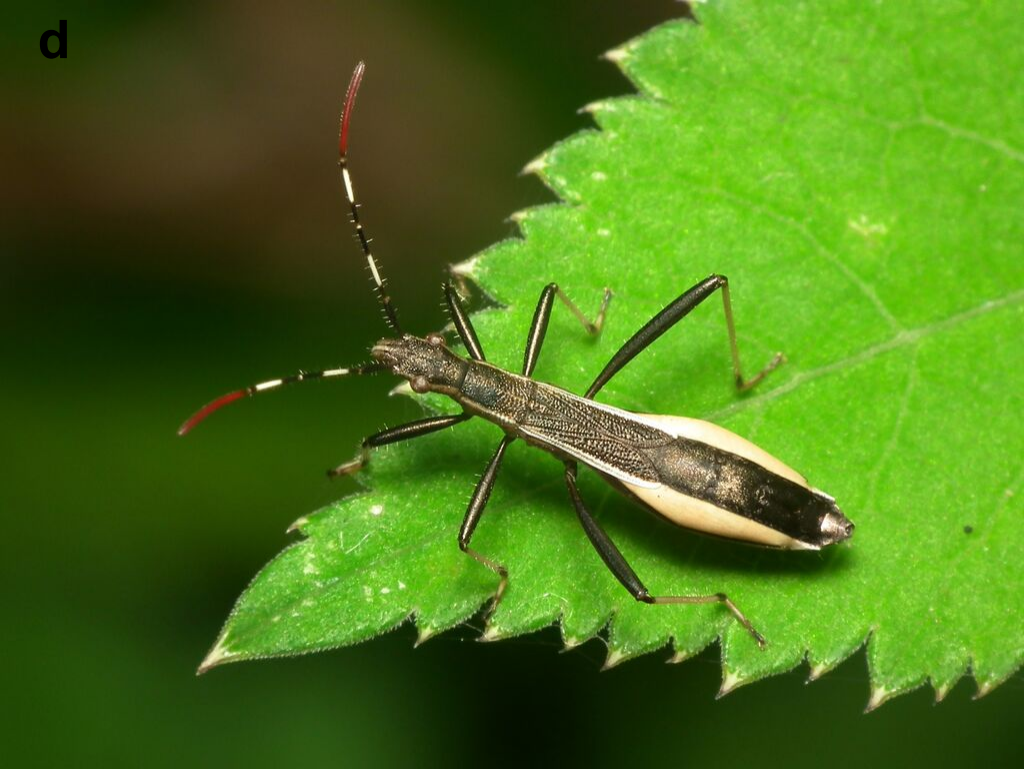
*Micrelytra fossularum* - BIN URI: BOLD:AEA8911

**Figure 1e.**
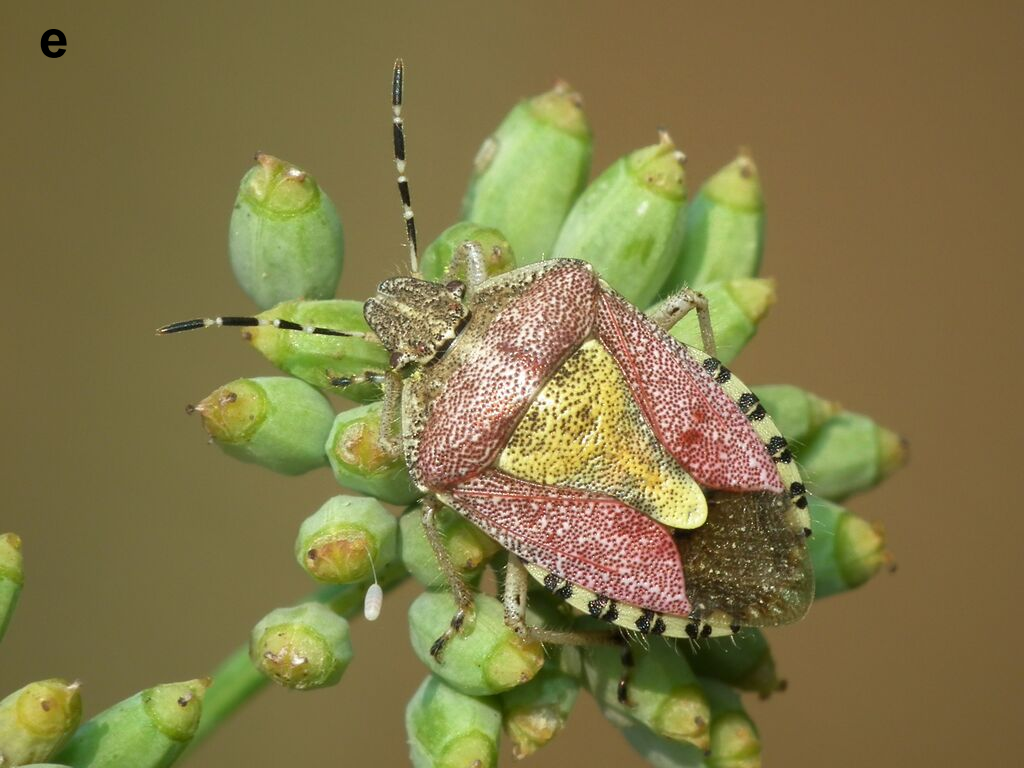
*Dolycoris baccarum* - BIN URI: BOLD:AAP3525

**Figure 1f.**
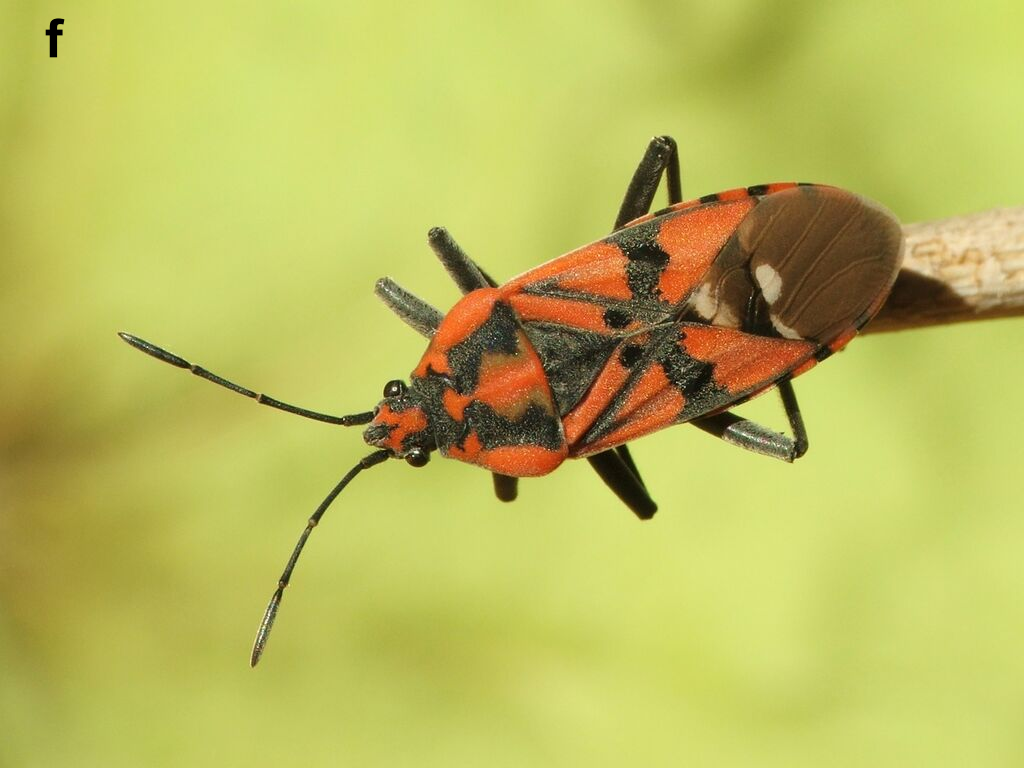
*Spilostethus pandurus* - BIN URI: BOLD:AAV0102

**Table 1.** List of species that were collected and DNA barcoded within this project. In column Taxa: * - indicates taxa without a DNA barcode prior to this study; '' - indicates taxa with less than 10 sequences available prior to this study; ^#^ - indicates taxa that are important Portuguese records.

**Family**	**Taxa**	**IBI code**	**BOLD code**	**BOLD BIN**	**GenBank**
**Suborder Auchenorrhyncha**					
Aphrophoridae	*Aphrophora alni* (Fallén, 1805)	INV09012	IBIHP182-20	BOLD:AEE6925	MW535978
Aphrophoridae	*Aphrophora corticea* Germar, 1821''	INV06714, INV07137	IBIHP128-20, IBIHP141-20	BOLD:ACT0928	MW536000, MW535990
Aphrophoridae	*Philaenus spumarius* (Linnaeus, 1758)	INV00536, INV08398, INV08399, INV08417	IBIHP100-20, IBIHP076-20, IBIHP077-20, IBIHP078-20	BOLD:AAB1850	MW535975, MW536068, MW535998, MW535976
Cercopidae	*Cercopis intermedia* Kirschbaum, 1868''	INV02955, INV07443	IBIHP046-20, IBIHP068-20	BOLD:AEC5811	MW536014, MW536066
Cercopidae	*Haematoloma dorsata* (Ahrens, 1812)''	INV06363	IBIHP055-20	BOLD:ABV4901	MW535969
Cicadellidae	*Cicadella viridis* (Linnaeus, 1758)	INV00537, INV02935	IBIHP010-19, IBIHP019-19	BOLD:ACB8347	MW535965, MW536088
Cicadellidae	*Eupelix cuspidata* Fabricius, 1775''	INV08568	IBIHP179-20	BOLD:ADN9562	MW536080
Cicadellidae	*Eutettix variabilis* Hepner, 1942^#^	INV00888	IBIHP185-21	BOLD:AAV0162	MW536051
Cicadellidae	*Fieberiella florii* Stål, 1864^#^	INV06761	IBIHP191-21	BOLD:ACJ7053	MW536009
Cicadellidae	*Iassus lanio* Linnaeus, 1761''	INV00854	IBIHP107-20	BOLD:ABW6633	MW536053
Cicadellidae	*Tremulicerus fulgidus* (Fabricius, 1775)*	INV00544	IBIHP011-19	BOLD:ABX8897	MW536072
Cicadidae	*Tettigettalna estrellae* (Boulard, 1982)	INV05324	IBIHP117-20	BOLD:ACQ4286	MW536030
Delphacidae	*Laodelphax striatellus* (Fallén, 1826)	INV07220	IBIHP193-21	BOLD:ABY1518	MW536002
Dictyopharidae	*Almana longipes* (Dufour, 1849)*	INV02255	IBIHP109-20	BOLD:AEE5516	MW535980
Dictyopharidae	*Dictyophara europaea* (Linnaeus, 1767)	INV10744	IBIHP074-20	BOLD:ADJ8496	MW535967
Flatidae	*Siphanta acuta* (Walker, 1851)^#^	INV00638	IBIHP183-21	BOLD:AAJ7097	MW535981
Membracidae	*Centrotus cornutus* (Linnaeus, 1758)''	INV06292	IBIHP125-20	BOLD:ACP8681	MW536003
**Suborder Heteroptera**					
Acanthosomatidae	*Cyphostethus tristriatus* (Fabricius, 1787)	INV08561	IBIHP081-20	BOLD:ACX9740	MW536060
Acanthosomatidae	*Elasmostethus interstinctus* (Linnaeus, 1758)	INV01286, INV03659	IBIHP186-21, IBIHP050-20	BOLD:ABZ2225	MW536008, MW535966
Alydidae	*Camptopus lateralis* (Germar, 1817)''	INV06810	IBIHP134-20	BOLD:ACP6596	MW536041
Alydidae	*Micrelytra fossularum* (Rossi, 1790)''	INV10738	IBIHP072-20	BOLD:AEA8911	MW536052
Aradidae	*Aradus flavicornis* Dalman, 1823''	INV07327	IBIHP063-20	BOLD:ABW4545	MW536087
Berytidae	*Berytinus montivagus* (Meyer, 1841)''	INV00836	IBIHP106-20	BOLD:ACA7025	MW536032
Coreidae	*Centrocoris spiniger* (Fabricius, 1781)''	INV06382	IBIHP189-21	BOLD:AEF4063	MW536019
Coreidae	*Ceraleptus lividus* Stein, 1858	INV00158	IBIHP040-20	BOLD:ACA7307	MW535973
Coreidae	*Enoplops scapha* (Fabricius, 1794)	INV04363, INV06334	IBIHP052-20, IBIHP053-20	BOLD:ABW9378	MW535971, MW536013
Coreidae	*Haploprocta sulcicornis* (Fabricius, 1794)*	INV06214	IBIHP120-20	BOLD:AAZ9600	MW536044
Coreidae	*Syromastus rhombeus* (Linnaeus, 1767)	INV00189, INV07148	IBIHP003-19, IBIHP033-19	BOLD:ABX4334	MW536095, MW535972
Corixidae	*Corixa affinis* Leach, 1817	INV06766	IBIHP133-20	BOLD:ACY0615	MW535977
Corixidae	*Corixa punctata* (Illiger, 1807)	INV03620, INV06765	IBIHP187-21, IBIHP192-21	BOLD:ACB1799	MW536081, MW536011
Corixidae	*Hesperocorixa sahlbergi* (Fieber, 1848)	INV06758	IBIHP131-20	BOLD:AAN0795	MW535983
Corixidae	*Paracorixa concinna* (Fieber, 1848)	INV00874	IBIHP184-21	BOLD:ADG5371	MW536062
Corixidae	*Sigara nigrolineata* (Fieber, 1848)	INV03590	IBIHP116-20	BOLD:ACB1978	MW536074
Corixidae	*Sigara venusta* (Douglas & Scott, 1869)''	INV00312	IBIHP005-19	BOLD:ABA5309	MW535962
Cydnidae	*Cydnus aterrimus* (Forster, 1771)''	INV00179	IBIHP002-19	BOLD:ABX7003	MW536015
Cydnidae	*Macroscytus brunneus* (Fabricius, 1803)''	INV07326, INV07329, INV08565	IBIHP144-20, IBIHP145-20, IBIHP082-20	BOLD:ADX9400	MW536058, MW535979, MW536077
Gerridae	*Aquarius najas* (DeGeer, 1773)	INV00319	IBIHP042-20	BOLD:AAN1521	MW536079
Gerridae	*Aquarius paludum* (Fabricius, 1794)	INV00384	IBIHP008-19	BOLD:AAI7450	MW536075
Gerridae	*Gerris gibbifer* Schummel, 1832	INV00354, INV00399	IBIHP006-19, IBIHP009-19	BOLD:ACB1756	MW536050, MW536031
Hydrometridae	*Hydrometra stagnorum* (Linnaeus, 1758)	INV00415	IBIHP043-20	BOLD:AEC2693	MW535986
Lygaeidae	*Lygaeus equestris* (Linnaeus, 1758)	INV06335, INV07424	IBIHP054-20, IBIHP066-20	BOLD:ACB9437	MW536020, MW535992
Lygaeidae	*Melanocoryphus albomaculatus* Goeze, 1778''	INV02909	IBIHP112-20	BOLD:AEE6008	MW536033
Lygaeidae	*Spilostethus pandurus* Scopoli, 1763	INV03741	IBIHP025-19	BOLD:AAV0102	MW536010
Lygaeidae	*Spilostethus saxatilis* (Scopoli, 1763)	INV00147	IBIHP001-19	BOLD:ADS4825	MW536006
Miridae	*Capsodes flavomarginatus* (Donovan, 1798)''	INV07922	IBIHP168-20	BOLD:ACR3434	MW536045
Miridae	*Closterotomus trivialis* (A.Costa, 1853)''	INV08815	IBIHP089-20	BOLD:AEA3807	MW536038
Miridae	*Harpocera thoracica* (Fallén, 1807)	INV08427	IBIHP080-20	BOLD:ABU6305	MW536078
Miridae	*Heterocordylus tibialis* (Hahn, 1833)	INV07441, INV07442	IBIHP152-20, IBIHP195-21	BOLD:ADM8543	MW536054, MW535993
Miridae	*Liocoris tripustulatus* (Fabricius, 1781)	INV10737	IBIHP071-20	BOLD:AAY9524	MW536005
Miridae	*Phytocoris varipes* Boheman, 1852	INV08602	IBIHP086-20	BOLD:AAH9369	MW535974
Miridae	*Psallus ambiguus* (Fallén, 1807)	INV07923	IBIHP169-20	BOLD:AAY8936	MW536057
Miridae	*Psallus varians* (Herrich-Schäffer, 1841)	INV08423	IBIHP079-20	BOLD:AAY8935	MW536027
Miridae	*Stenodema laevigata* (Linnaeus, 1758)	INV06213, INV07428	IBIHP119-20, IBIHP150-20	BOLD:AAY9089	MW535964, MW536092
Nabidae	*Himacerus mirmicoides* (O.Costa, 1834)	INV02906	IBIHP111-20	BOLD:AAY9075	MW536034
Nepidae	*Nepa cinerea* Linnaeus, 1758	INV00165, INV00628, INV02496	IBIHP041-20, IBIHP044-20, IBIHP045-20	BOLD:AEC3215	MW536073, MW535968, MW535999
Nepidae	*Ranatra linearis* (Linnaeus, 1758)	INV00744, INV00745, INV03529, INV03530, INV03531	IBIHP016-19, IBIHP017-19, IBIHP020-19, IBIHP021-19, IBIHP022-19	BOLD:AAL1328	MW535994, MW536004, MW536067, MW535995, MW536048
Notonectidae	*Anisops sardeus* Herrich-Schäffer, 1850''	INV03534	IBIHP114-20	BOLD:ABV0079	MW536012
Notonectidae	*Notonecta maculata* Fabricius, 1794	INV00310, INV00378, INV00681, INV03281	IBIHP004-19, IBIHP007-19, IBIHP015-19, IBIHP048-20	BOLD:AAN1703	MW536040, MW536091, MW536069, MW536061
Pentatomidae	*Aelia acuminata* (Linnaeus, 1758)	INV06436	IBIHP056-20	BOLD:AAY9083	MW536042
Pentatomidae	*Aelia rostrata* Boheman, 1852''	INV02960, INV03567, INV06812	IBIHP113-20, IBIHP115-20, IBIHP135-20	BOLD:AEE2078	MW536063, MW536076, MW536096
Pentatomidae	*Carpocoris mediterraneus atlanticus* Tamanini, 1959''	INV06846, INV00523	IBIHP060-20, IBIHP099-20	BOLD:ACD7499	MW536043, MW536029
		INV10749	IBIHP075-20	BOLD:ACD6303	MW536064
Pentatomidae	*Dolycoris baccarum* (Linnaeus, 1758)	INV07921	IBIHP036-19	BOLD:AAP3525	MW536086
Pentatomidae	*Eurydema ornata* (Linnaeus, 1758)	INV06849	IBIHP061-20	BOLD:AEC2842	MW536016
		INV07035	IBIHP139-20	BOLD:AEE5125	MW536022
Pentatomidae	*Eysarcoris venustissimus* (Schrank, 1776)''	INV06293, INV06337, INV07432	IBIHP030-19, IBIHP031-19, IBIHP035-19	BOLD:ADZ2301	MW536070, MW536026, MW536007
Pentatomidae	*Graphosoma italicum* (O.F.Müller, 1766)	INV03552, INV07430, INV08816	IBIHP049-20,IBIHP034-19, IBIHP090-20	BOLD:AAY9133	MW536059, MW536036, MW536094
Pentatomidae	*Graphosoma semipunctatum* (Fabricius, 1775)	INV00620	IBIHP013-19	BOLD:ADT8242	MW536018
Pentatomidae	*Holcogaster fibulata* (Germar, 1831)''	INV07688	IBIHP069-20	BOLD:ACS3305	MW535988
Pentatomidae	*Nezara viridula* (Linnaeus, 1758)	INV00142, INV03969, INV04120	IBIHP039-20, IBIHP026-19, IBIHP028-19	BOLD:AAU3346	MW535996, MW536046, MW536001
Pentatomidae	*Palomena prasina* (Linnaeus, 1761)	INV00676, INV07422	IBIHP014-19, IBIHP065-20	BOLD:AAG8727	MW536049, MW536037
Pentatomidae	*Pentatoma rufipes* (Linnaeus, 1758)	INV04074, INV10253	IBIHP027-19, IBIHP070-20	BOLD:AAZ7767	MW536047, MW536039
Pentatomidae	*Piezodorus lituratus* (Fabricius, 1794)	INV08914	IBIHP091-20	BOLD:AAY9491	MW536093
Pentatomidae	*Rhaphigaster nebulosa* (Poda, 1761)	INV03554	IBIHP023-19	BOLD:AAY8964	MW536089
Potamocoridae	*Naucoris maculatus* Fabricius, 1798''	INV00631	IBIHP104-20	BOLD:AEE8147	MW536056
Pyrrhocoridae	*Pyrrhocoris apterus* (Linnaeus, 1758)	INV01258, INV06470	IBIHP018-19, IBIHP032-19	BOLD:AAY8951	MW536084, MW535963
Pyrrhocoridae	*Scantius aegyptius* (Linnaeus, 1758)''	INV04125	IBIHP051-20	BOLD:ACL0625	MW536071
Reduviidae	*Empicoris rubromaculatus* (Blackburn, 1888)	INV07642	IBIHP164-20	BOLD:ACN7256	MW536083
Reduviidae	*Oncocephalus gularis* Reuter, 1882*	INV08587	IBIHP085-20	BOLD:AEH0127	MW535991
Reduviidae	*Reduvius personatus* (Linnaeus, 1758)	INV06813	IBIHP059-20	BOLD:AEC5973	MW536035
		INV08753	IBIHP088-20	BOLD:AEE8716	MW536025
Reduviidae	*Rhynocoris cuspidatus* Ribaut, 1922*	INV02959	IBIHP047-20	BOLD:ADJ4404	MW535984
Rhopalidae	*Brachycarenus tigrinus* (Schilling, 1829)	INV00545	IBIHP012-19	BOLD:AAD4531	MW536017
Rhopalidae	*Chorosoma schillingii* (Schilling, 1829)	INV02601, INV03608, INV06759	IBIHP029-19, IBIHP024-19, IBIHP057-20	BOLD:ABV9616	MW536028, MW535987, MW536082
Rhopalidae	*Liorhyssus hyalinus* (Fabricius, 1794)	INV08920	IBIHP092-20	BOLD:AAG8881	MW536021
Rhopalidae	*Rhopalus subrufus* (Gmelin, 1790)	INV07425	IBIHP067-20	BOLD:AAY9322	MW536085
Rhopalidae	*Stictopleurus punctatonervosus* (Goeze, 1778)^#^	INV10743	IBIHP073-20	BOLD:AAZ3560	MW535985
Rhyparochromidae	*Beosus maritimus* (Scopoli, 1763)	INV07290	IBIHP062-20	BOLD:ABW9272	MW536023
Rhyparochromidae	*Eremocoris fenestratus* (Herrich-Schäffer, 1839)*	INV06764	IBIHP058-20	BOLD:ABU6590	MW535989
Stenocephalidae	*Dicranocephalus agilis* (Scopoli, 1763)''	INV07328	IBIHP064-20	BOLD:ADK7724	MW536090
Thaumastocoridae	*Thaumastocoris peregrinus* Carpintero & Dellapé, 2006	INV07942, INV07943	IBIHP037-19, IBIHP038-19	BOLD:ACY9011	MW535982, MW535970

**Table 2.** Number of specimens and species collected per Portuguese District and corresponding percentage.

**District**	**Specimens (n)**	**Specimens (%)**	**Taxa (n)**	**Taxa (%)**
Bragança	72	55.0%	55	62.5%
Porto	13	9.9%	13	14.8%
Guarda	10	7.6%	9	10.2%
Setúbal	7	5.3%	7	8.0%
Aveiro	5	3.8%	4	4.5%
Leiria	5	3.8%	3	3.4%
Castelo Branco	4	3.1%	3	3.4%
Faro	4	3.1%	2	2.3%
Lisboa	3	2.3%	3	3.4%
Vila Real	3	2.3%	3	3.4%
Viseu	2	1.5%	2	2.3%
Beja	1	0.8%	1	1.1%
not known	2	1.5%	2	2.3%
**TOTAL**	131		88	
